# The Role of Serotonin in the Influence of Intense Locomotion on the Behavior Under Uncertainty in the Mollusk *Lymnaea stagnalis*

**DOI:** 10.3389/fphys.2020.00221

**Published:** 2020-03-17

**Authors:** Hitoshi Aonuma, Maxim Mezheritskiy, Boris Boldyshev, Yuki Totani, Dmitry Vorontsov, Igor Zakharov, Etsuro Ito, Varvara Dyakonova

**Affiliations:** ^1^Research Center of Mathematics for Social Creativity, Research Institute for Electronic Science, Hokkaido University, Hokkaido, Japan; ^2^Core Research for Evolutional Science and Technology (CREST), Japan Science and Technology Agency, Saitama, Japan; ^3^Koltzov Institute of Developmental Biology of the Russian Academy of Sciences (RAS), Moscow, Russia; ^4^Trapeznikov Institute of Control Sciences of Russian Academy of Sciences (RAS), Moscow, Russia; ^5^Department of Biology, Waseda University, Tokyo, Japan

**Keywords:** serotonin, decision-making, mollusks, effect of exercise on the brain, goal-directed locomotion

## Abstract

The role of serotonin in the immediate and delayed influence of physical exercise on brain functions has been intensively studied in mammals. Recently, immediate effects of intense locomotion on the decision-making under uncertainty were reported in the Great Pond snail, *Lymnaea stagnalis* (Korshunova et al., 2016). In this animal, serotonergic neurons control locomotion, and serotonin modulates many processes underlying behavior, including cognitive ones (memory and learning). Whether serotonin mediates the behavioral effects of intense locomotion in mollusks, as it does in vertebrates, remains unknown. Here, the delayed facilitating effects of intense locomotion on the decision-making in the novel environment are described in *Lymnaea*. Past exercise was found to alter the metabolism of serotonin, namely the content of serotonin precursor and its catabolites in the cerebral and pedal ganglia, as measured by high-performance liquid chromatography. The immediate and delayed effects of exercise on serotonin metabolism were different. Moreover, serotonin metabolism was regulated differently in different ganglia. Pharmacological manipulations of the serotonin content and receptor availability suggests that serotonin is likely to be responsible for the locomotor acceleration in the test of decision-making under uncertainty performed after exercise. However, the exercise-induced facilitation of decision-making (manifested in a reduced number of turns during the orienting behavior) cannot be attributed to the effects of serotonin.

## Introduction

The influence of physical exercise on brain function has been thoroughly investigated in various mammalian species (van Praag et al., 1999; Salmon, 2001; Cotman et al., 2007; Hillman et al., 2008; Roig et al., 2012; Laurence et al., 2015). It has been suggested that feedforward brain activation caused by intense locomotion is a widespread phenomenon throughout the animal kingdom that may be especially beneficial for subsequent orientation and adaptation in the novel environment (Stevenson et al., 2005; Dyakonova and Krushinsky, 2008; Korshunova et al., 2016).

In mammals, physical activity affects neuromodulatory and neurotrophic systems (Heijnen et al., 2016), namely, the serotonergic and dopaminergic systems (Kondo and Shimada, 2015; Heijnen et al., 2016), endogenous opioid and cannabinoid systems (Heijnen et al., 2016), brain-derived neurotrophic factor (BDNF) (Vaynman et al., 2004), insulin-like growth factor-1 (Carro et al., 2001), and other factors and neuromodulators (Heijnen et al., 2016). The increased release of these factors activates neurogenesis in the hippocampus (van Praag et al., 1999, Lee et al., 2013). The later had been considered a key factor for improved cognitive performance after exercise. However, stimulation of neurogenesis alone was recently found to be insufficient to reproduce the behavioral effects of exercise in rodents (Choi et al., 2018). Activation of BDNF synthesis and release, known to have strong interactions with the serotonergic system (Popova et al., 2017), is required to mimic these effects (Choi et al., 2018). Strong effects of intense locomotion on subsequent behavior are reported in a distantly related group of animals, the protostomes (Hofmann and Stevenson, 2000; Dyakonova and Krushinsky, 2008; Korshunova et al., 2016), suggesting that mechanisms other than augmentation of hippocampal neurogenesis can mediate the behavioral benefits of exercise.

Previously, we reported that the behavioral effects of intense locomotion in the *Lymnaea stagnalis* snail are, in many aspects, similar to those observed in rodents and humans, with a decrease in defensive responses, an increase in general activity (D’yakonova, 2014) and a facilitation of decision-making in the novel environment (Korshunova et al., 2016). Terrestrial-like crawling in low water for 2 h prior to the test promoted the transition from slow circular locomotion to the fast goal-oriented crawl in asymmetrically lit arena (Korshunova et al., 2016). We concluded that exercise “facilitates the transition from uncertainty to decision-making in snails.”

Serotonin activates many forms of behavior (Sakharov, 1990; Gillette, 2006) and participates in the regulation of cognitive functions, such as learning and memory, in mollusks (Balaban et al., 2016; Nikitin et al., 2018; Totani et al., 2019). The neurons that control locomotion in *L. stagnalis* include a large serotonergic PeA cluster of cells which release serotonin to the muscles and cilia in the sole (Syed and Winlow, 1989; Longley and Peterman, 2013). The PeA neurons have also been suggested to have a neuromodulatory and neurohormonal role in the central nervous system (Chistopol’skii and Sakharov, 2003; Dyakonova et al., 2015). Many of the above-mentioned behavioral effects of exercise are similar to the influences ascribed to the increased level of serotonin in *Lymnaea*. Therefore, we hypothesized that the increased activity of these neurons is required for intense locomotion (Dyakonova et al., 2019) and that enhanced extrasynaptic release of serotonin may underlie the behavioral changes observed after the motor load.

The aim of the present study was to elucidate possible involvement of serotonin in the regulation of behavior in the novel environment and in the effects of intense locomotion on this behavior. Specifically, we addressed the following questions: (1) whether the behavioral effects of the exercise persist for 2 h after its termination; (2) what metabolic changes in the serotonergic system accompany intense locomotion and recovery; and (3) how serotonergic ligands influence the above-described behavior of *Lymnaea* in the novel environment. The obtained data suggest that serotonin is involved in the mediation of motor arousal in the novel environment. At the same time, serotonin is not responsible for faster decision-making observed after exercise. It is likely that along with serotonin, other factors participate in the mechanism of the exercise effect on decision-making.

## Materials and Methods

### Animals

The *Lymnaea stagnalis* snails were taken from a breeding colony initially obtained from the Netherlands (1992) and mixed with specimen from wild population of the Oka River, Moscow region, Russia. They were fed daily on lettuce *ad libitum* and kept in dechlorinated tap water at room temperature.

### Forced Locomotion in Low Water (Experiments 1 and 2)

Enhanced motor activity was evoked (as in Korshunova et al., 2016) by putting the snails into a tank (50 × 50 cm) filled with 1–2 mm layer of water for 2 or 4 h. As was reported earlier this procedure “protected snails from drying but forced them to use intense muscular crawling to compensate for the lack of water supporting the weight of their shells” (Korshunova et al., 2016, Figure 1A). Control snails were kept in deep water so they could use ciliary locomotion in similar light conditions. In addition, the control group was divided into the snails taken directly from their breeding colony (Ch) and the snails that were kept for 2 h in 300 ml containers filled with the same water as in the breeding stock (Cn). The Ch group was used to control whether novelty by itself produces locomotor arousal in snails. Rest after exercise was evoked by putting snails into a cylinder filled with water up to 9 cm for 2 h after intense terrestrial motor activity in low water. The experimental and control animals were investigated in random order in a single experiment.

**FIGURE 1:**
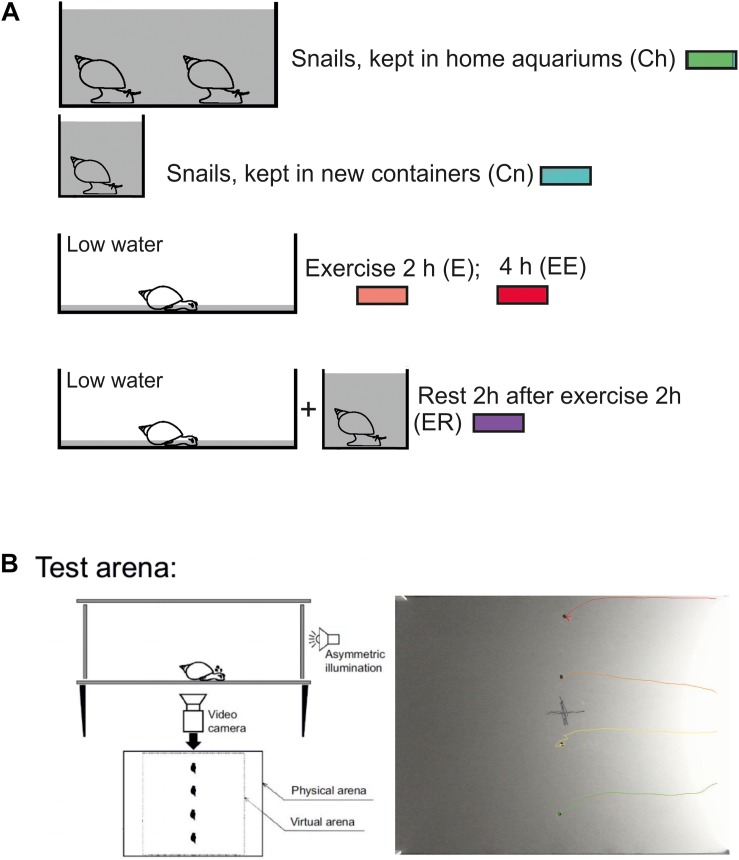
Schematic representation of the experimental procedure. **(A)** Procedure for the investigation of the effects of enhanced motor activity (exercise, E, EE) and rest after exercise (ER), redrawn after (Korshunova et al., 2016; Dyakonova et al., 2019). In double-blind experiments, snails were divided into control and experimental groups and resided in similar light conditions before being placed in the test arena. Control snails were kept in deep water so they could use ciliary locomotion for 2 h in similar light conditions. Two control groups were used: snails taken directly from their breeding colony (Ch, marked with green color) and snails that were kept for 2 h in 300 ml containers filled with the same water as in the breeding stock (Cn, marked with blue color). The last group was used to test whether novelty by itself produces locomotor arousal in snails. The experimental and control animals were investigated in parallel in one experiment. Snails of the ‘exercise’ experimental group (E, marked with red color) were kept in a tank (25 × 50 cm) filled with a thin (1 mm) layer of water which protected them from drying but forced them to use crawling (intense muscular locomotion) to compensate for the lack of water supporting the weight of the shell for 2 h (E, marked with light red color) or 4 h (EE, marked with dark red color). Snails of the “rest after exercise” experimental group (ER, marked with purple color) were placed into in a cylinder filled with water up to 9 cm to be able to use ciliary locomotion for 2 h after 2 h of exercise in low water. In different experimental series, the combination of control and experimental groups could vary, for details see Methods and Results. **(B)** Behavioral paradigm for the investigation of decision-making in a novel environment. One or four snails (in different experimental series) were placed on the same central points of a square arena on a flat and dry plastic surface with one-side asymmetric illumination. All snail movements were tracked and video-recorded for 15 min in each experiment. The camera was placed under the transparent plastic bottom of the arena. For the behavioral analysis, the virtual arena border (22 cm from the central point) was defined. C, example of one record from four simultaneously tracked snails. In their behavior, the two clearly distinct phases can be seen: (1) uncertain movements, characterized by comparatively low speeds of movement, stops and repeated changes of movement direction and (2) an intense fast locomotion in a chosen direction as previously reported by Korshunova et al. (2016).

### Test for Behavior in the Novel Environment (Experiment 1)

We used the same test and set up as in Korshunova et al. (2016). Individual snails (one or four at a time in different experiments) were placed into a rectangular arena (60 × 45 cm) on a flat dry plastic surface (Figure 1B). One of the shorter walls of the arena was made of transparent plastic to provide the asymmetric illumination. All other walls were covered with non-transparent black film. A 40W white light bulb placed at a distance outside the arena served as a source of light. The light intensity at the arena’s center (measured 3cm above the surface with the Proskit MT-4017 luxmeter) was adjusted to 30 lux, and it was 83 and 12 lux near the light and the dark walls, respectively. No measurable amount of light came to the arena from below. We did not attempt to avoid temperature gradient (less than 1°C, measured with a mini-infrared thermometer) caused by the light source in order to maintain natural conditions in which more lighted areas are typically warmer.

The movements of each snail were recorded at 15 frames per second for 15 min with the Microsoft LifeCam HD-3000 video camera. The recordings were video-tracked using the EthoVision XT software (Noldus, the Netherlands) and independently scored manually with RealTimer (Openscience, Russia). The camera was placed under the transparent plastic bottom of the arena. The traces left by the crawling snails were removed with a clean paper towel before each new test.

During the analysis, a centered square zone (22 × 22 cm) that limited the track and scoring analysis was defined (Figure 1B). This zone was used to exclude from the analysis snail’s movements near the physical borders of the arena, especially near the brightly lit wall. We evaluated (i) the time to the snail’s first movement, (ii) the total rotation prior to the crawling in the chosen direction, (iii) the mean velocity of locomotion (iv) the time taken to crawl to the virtual border of the arena. If the snail would not reach the virtual border, the time was given as the total time of observation, which was 15 min. Blind procedure was used for the tests performed manually (i, ii).

### Measurement of 5-HT and Its Metabolites in the Pedal and Cerebral Ganglia of *Lymnaea* (Experiment 2)

5-HT and its precursor and catabolites were measured as described previously (Aonuma and Watanabe, 2012; Aonuma et al., 2016, 2017, 2018). Briefly, snails were quickly frozen using liquid N_2_, and the pedal and cerebral ganglia were dissected out of the ice-cold *Lymnaea* physiological solution. Each paired ganglion was homogenized in 50 μl ice-cold 0.1M perchloric acid containing 5 ng of N-ω-methyl-5-hydroxytryptamine oxalate (NMET; Sigma-Aldrich, St. Louis, MO, United States) as an internal standard. After centrifugation of the homogenate (at 0°C, 21.500 g (15.000 rpm), for 30 min), 40 μl of supernatant was collected. Using high-performance liquid chromatography with electrochemical detection (HPLC-ECD; EICOM, Kyoto, Japan), we measured the following compounds in each of the four groups: (1) serotonin (5-HT); (2) a precursor of 5-HT (5-hydroxytryptophan, 5-HTP); and two catabolites of 5-HT, (3) N-acetylserotonin (Nac-5-HT) and (4) 5-hydroxyindole acetaldehyde (5-HIAA). The mobile phase containing 0.18M chloroacetic acid and 16 μM disodium EDTA was adjusted to pH3.6 with NaOH. Sodium-1-octanesulfonate at 1.85 mM as an ion-pair reagent and acetonitrile (CH3CN) at 8.40% (v/v) as an organic modifier were added to the mobile phase solution. The supernatants of samples were injected directly onto the HPLC column (C18 reversed-phase column, CAPCELL PAK C18MG, Shiseido, Tokyo, Japan) heated to 30°C in a column oven. A glass carbon electrode (WE-GC, EICOM Co.) was used for electrochemical detection (ECD-100, EICOM Co.). The detector potential was set at 890 mV versus an Ag/AgCl reference electrode. The chromatographs were acquired using the computer program PowerChrom (eDAQ Pty, Denistone East, NSW, Australia).

### Drug Administration (Experiments 3, 4, 5)

Two different procedures of drug administration were used: (1) immersion of snails in groups of four (5-HT experiment) or individually (5-HTP experiment) into water (300 ml) taken from the breeding stock, with the drug added, for 2 h prior to the test, or (2) injection of 100 μl physiological solution (50 mM NaCl, 1.6 mM KCl, 4 mM CaCl_2_, 8 mM MgCl_2_, 10 mM Tris, pH7.6) containing the drug (ketanserin) 15 min prior to the test. The control snails for the experiments with 5-HTP and 5-HT were placed into the 300 ml containers filled with water taken from the breeding stock for 2 h. Snails injected with 100 μl vehicle (physiological solution) comprised the control group in the experiment with the serotonin antagonist ketanserin. The drugs were administered in the following concentrations: 5-HTP (0.1 mM), 5-HT (0.1 mM), ketanserin (0.02 and 0.1 mÌ). All drugs were obtained from Sigma Aldrich (Moscow, Russia). The test procedure was the same as in Experiment 1 (decision-making in the novel environment).

### Data Analysis

The significance of the differences was tested by the non-parametric Mann–Whitney *U*-test for experiments with one control and one experimental group. The non-parametric Kruskal-Wallis test for multiple comparisons with *post hoc* comparisons was applied for three and more groups investigated in one experiment. The analysis was performed using the STATISTICA software (Statsoft). The values presented in the figures are given as the median with upper and lower quartiles.

## Results

### The Delayed Effects of Intense Locomotion on Decision-Making (Experiment 1)

In mammals, exercise has both immediate and long-term cognitive effects, which recruits different mechanisms (Heijnen et al., 2016). We have demonstrated earlier that 2 h of muscular crawling in *Lymnaea* results in the facilitation of decision-making and acceleration of locomotion when snails are placed in the novel environment (Korshunova et al., 2016). Here, we tested whether or not the exercise has any delayed effects following 2 h of rest.

The snails that rested in 300 ml aquatic containers filled with the water taken from the breeding stock (*n* = 31, ER) for 2 h after exercise, control snails taken directly from their breeding colony (*n* = 30, Ch) and control snails that resided in 300 ml containers for 2 h (*n* = 30, Cn) were used as three groups in this experiment. The third group (Cn, snails kept in novel containers) was used since the novelty by itself produces locomotor arousal in snails.

**FIGURE 2:**
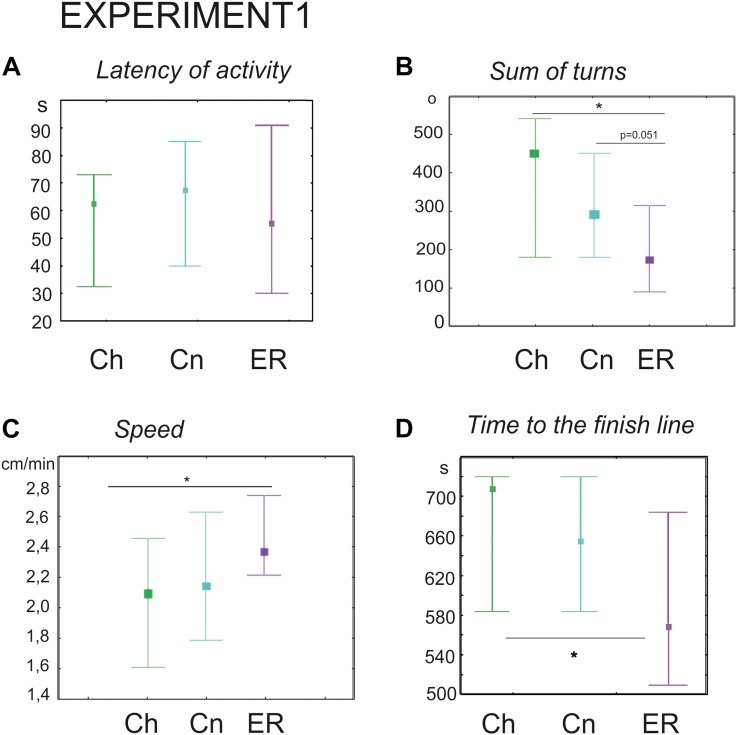
Effects of novelty and “exercise then rest” on the behavior of snails on a dry surface. **(A)** The latency of the first movement in the new environment, in seconds. **(B)** The sum of turns prior to decision-making, in degrees. **(C)** The speed of locomotion, cm/min. **(D)** Time to the finish line (virtual arena borders), in seconds. Left to right in each diagram: green color indicates snails that were taken from the breeding stock (Ch, *n* = 30); blue color indicates snails that were kept for 2 h in a cylinder filled with water up to 9 cm (Cn, *n* = 30); purple color indicates snails that were placed for 2 h in low water to exercise and then rested for 2 h in a container filled with water up to 9 cm (ER, *n* = 31); The significance of differences was tested using the multiple comparisons Kruskal–Wallis test, followed by *post hoc* tests. All values are given as median with the lower and upper quartiles * indicates *p* < 0.05.

**FIGURE 3:**
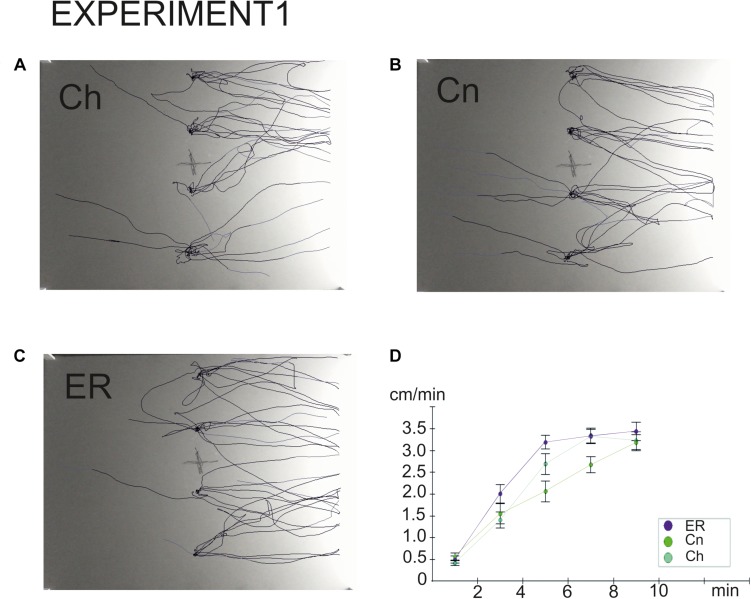
Overlay tracks of snails the locomotor arousal in control and “exercise then rest.” **(A)** Snails that were taken from the breeding colony (Ch). **(B)** Snails that were kept for 2 h in a cylinder filled with water up to 9 cm (Cn). **(C)** Snails that were placed for 2 h in low water to exercise and then rested for 2 h in a container filled with water up to 9cm (ER). Tracks presented in C show that ER snails spent less time in the central zone and made fewer turns than Ch and Cn snails although the dark/light choice was not different between Ch, Cn, and ER. In **(B)**, the effect of novelty can be seen as well, the rotational behavior is weaker in **(B)** than in **(A)**. **(D)** Locomotor arousal in the Ch, Cn, and ER groups: the mean speed of locomotion by equal time intervals (1–2 min, 2–4 min, 4–6 min, 6–8 min, 8–10 min).

The latency of the first movement was no different (Kruskal–Wallis test: *H* = 0.28, *p* = 0.8), however, the ER snails made fewer turns prior to decision-making (Kruskal–Wallis test: H (2, *N* = 91) = 13.01, *p* = 0.0015; Figures 2B, 3A–C, *post hoc* tests ER/Ch *p* = 0.0013; ER/Cn *p* = 0.051; Ch/Cn *p* = 0.79) and had higher speed of locomotion compared to the control group taken from the breeding stock (Kruskal–Wallis test: H (2, *N* = 91) = 7.5, *p* = 0.0234; Figure 2C, *post hoc* tests ER/Ch *p* = 0.020; ER/Cn *p* = 0.2; Ch/Cn *p* = 0.9). Recently, we reported that locomotor arousal developed more quickly in the ER snails than in the Cn group (Dyakonova et al., 2019). Similar trend can be seen in Figure 3D. **H**owever, there was no significant difference in the mean speed between the ER and the Cn group. The ER snails reached and crossed the virtual border faster than the Ch snails (Kruskal–Wallis test: H (2, *N* = 91) = 9.1, *p* = 0.010, Figure 2D, *post hoc* tests ER/Ch *p* = 0.011; ER/Cn p = 0.1; Ch/Cn *p* = 0.9). The ratio of light/dark choice was approximately the same in all groups ca. 75/25% (Figures 3–C). This ratio corresponded to the one reported earlier (Korshunova et al., 2016). No cases of intermediate choice were observed.

We performed an additional set of experiments with only one control group kept in novel conditions (Cn, *n* = 65) to validate the delayed effect of exercise (ER, *n* = 62) on the performance of turns. ER snails made significantly fewer turns (181 ± 17 versus 258 ± 22 degrees in the Cn, Mann–Whitney *U*-test, *z* = 2.7; *p* = 0.0058, not illustrated).

Therefore, exercise followed by rest decreased the display of “uncertainty” in the snail behavior in the novel environment, promoting the switch from rotational behavior to decision-making. Preconditioning in novel containers has not induced significant behavioral effects in comparison to intact control (Cn versus Ch).

### Immediate and Delayed Effects of Intense Locomotion on the Serotonin Content Within the Pedal and Cerebral Ganglia of *Lymnaea* (Experiment 2)

Four separate groups of snails were used (Figure 1A): (1) control snails taken from aquatic conditions (Cn); (2) snails that had been crawling (exercising) for 2 h in low water (E); (3) snails that had been “resting” in deep water for 2 h after 2 h of exercise (ER); and (4) snails that had been crawling (exercising) for 4 h in low water (EE). We measured the levels of 5-HTP, 5-HT, Nac-5-HT, and 5-HIAA in the in the pedal and cerebral ganglia. The pedal ganglia contain neurons related to locomotion, including the serotonergic PeA cluster, whose activity is influenced by intense locomotion (Korshunova et al., 2016; Dyakonova et al., 2019). The cerebral ganglia were included in the analysis for two reasons: first, to verify the hypothesis that serotonin metabolism in the functionally distinct parts of the CNS can be differently affected by the same behavioral context; second, to check whether the exercise produces any changes in the serotonin metabolism within the “cognitive” (cerebral) ganglia of the snail CNS that are believed to participate in learning, memory and decision-making.

#### Pedal Ganglia

**FIGURE 4:**
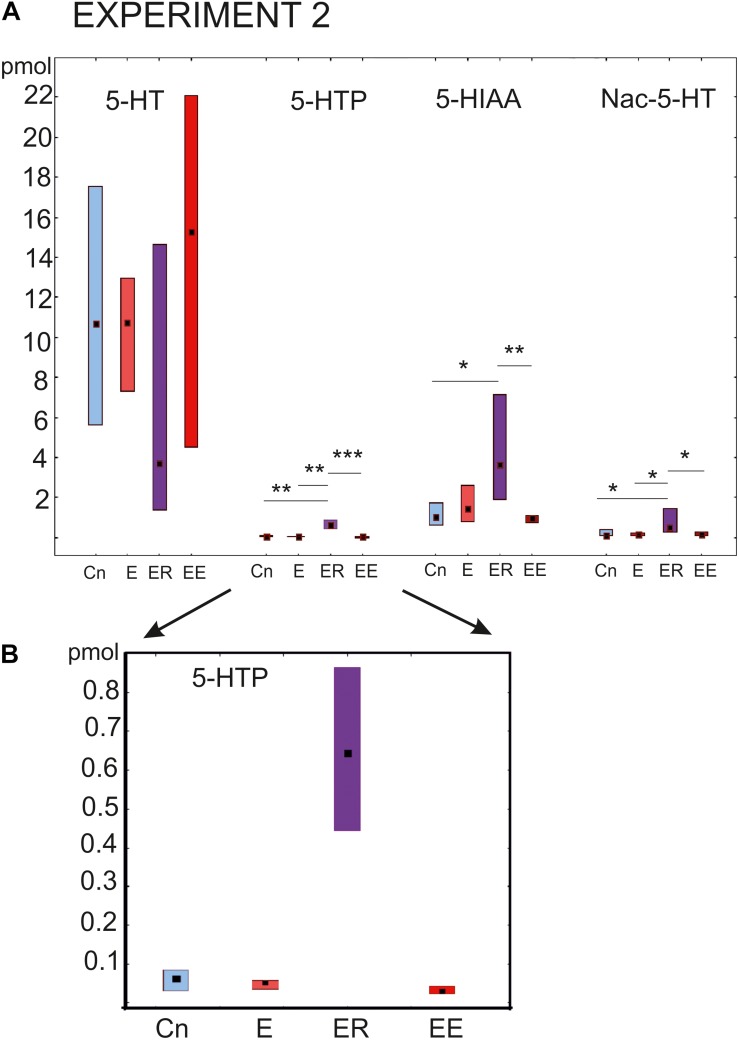
Acute and delayed effects of intense locomotion on the content of serotonin and its catabolites within the pedal ganglia of *Lymnaea* measured with high-performance liquid chromatography with electrochemical detection (pmol). **(A)** Left to right: Serotonin (5-HT); a precursor of 5-HT (5-hydroxytryptophan, 5-HTP, see also below in a higher scale); N-acetylserotonin, Nac-5-HT; 5-hydroxyindole acetoaldehyde, 5-HIAA. **(B)** The magnified scale for 5-HTP content shown in **(A)** second to the left. Blue color indicates control snails that were kept in a new cylinder filled with water up to 9 cm for 2 h (Cn, *n* = 13); light red color indicates snails that were placed for 2 h in low water to exercise (E, *n* = 11); purple color indicates snails that were placed for 2 h in low water to exercise and then rested for 2 h in a container filled with water up to 9 cm (ER, *n* = 13); dark red color indicates snails that were placed for 4 h in low water to exercise (EE, *n* = 12). The significance of differences was tested using the multiple comparisons Kruskal-Wallis test, followed by *post hoc* tests. All values are given as median with the lower and upper quartiles *, **, *** indicate *p* < 0.05, 00.1 and 0.001, respectively.

The most prominent changes in the content of the substances being investigated were observed in the group that rested after exercise (ER, Figure 4). The concentration of the serotonin precursor 5-HTP was more than 10 times higher (*p* < 0.001) than in all other groups (Kruskal–Wallis test: H (3, *N* = 46) = 29.16, *p* = 0.0001, Figure 4 *post hoc* tests ER/Cn *p* = 0.0016; ER/E *p* = 0.002; ER/EE *p* = 0.0001; E/Cn *p* = 0.9; EE/Cn *p* = 0.79).

Similarly, the 5-HIAA content was the highest in the ER group, with highly significant differences compared to the control and the 4 h -exercised (EE) snails (Kruskal-Wallis test: H (3, *N* = 46) = 13.08, *p* = 0.0045, Figure 4, the third group of bars, *post hoc* tests ER/Cn *p* = 0.029; ER/E *p* = 0.49; ER/EE *p* = 0.004; E/Cn *p* = 0.9; EE/Cn *p* = 0.85).

The Nac-5-HT content was significantly higher in the ER group compared to all other groups [Kruskal–Wallis test: H (3, *N* = 45) = 12.8, *p* = 0.0051, Figure 4, the last group of bars, *post hoc* tests ER/Cn *p* = 0.019; ER/E *p* = 0.04; ER/EE *p* = 0.01; E/Cn *p* = 0.9; EE/Cn *p* = 0.9].

There were no significant differences in the serotonin content [H (3, *N* = 47) = 4.07, *p* = 0.25]. In the ER group, serotonin showed the lowest concentration, but the tendency did not reach the level of significance (Figure 4, first group of bars). Neither 2 nor 4 h of intense locomotion resulted in any significant increase in the serotonin content within the pedal ganglia, although in the group exercised for 4 h, a trend of increased serotonin was observed but only achieved a p value equal to 0.1.

#### Cerebral Ganglia

**FIGURE 5:**
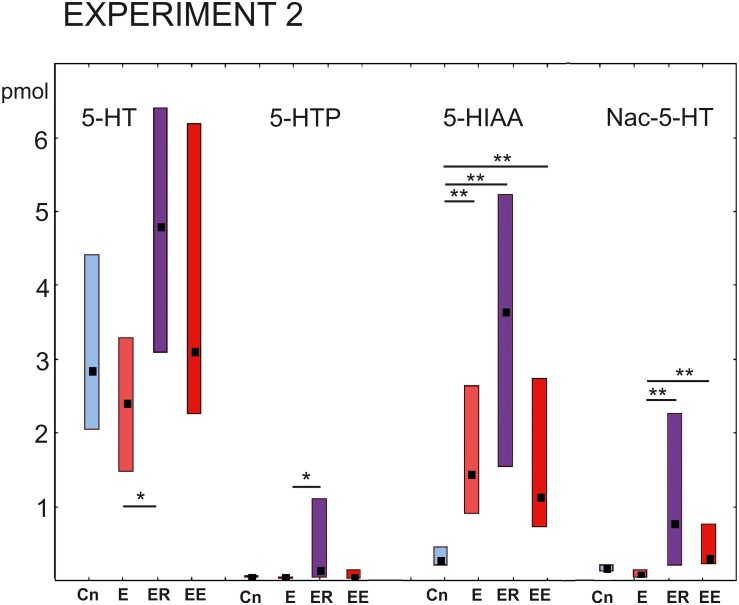
Acute and delayed effects of intense locomotion on the content of serotonin and its catabolites within the cerebral ganglia of *Lymnaea* measured with high-performance liquid chromatography with electrochemical detection (pmol). Left to right: Serotonin (5-HT); a precursor of 5-HT (5-hydroxytryptophan, 5-HTP); N-acetylserotonin, Nac-5-HT; 5-hydroxyindole acetoaldehyde, 5-HIAA. Blue color indicates control snails that were kept in a cylinder filled with water up to 9 cm for 2 h (Cn, *n* = 13); light red color indicates snails that were placed for 2 h in low water to exercise (E, *n* = 14); purple color indicates snails that were placed for 2 h in low water to exercise and then rested for 2 h in a container filled with water up to 9 cm (ER, *n* = 13); dark red color indicates snails that were placed for 4 h in low water to exercise (EE, *n* = 12). The significance of differences was tested using the multiple comparisons Kruskal-Wallis test, followed by *post hoc* tests. Values are given as median with the lower and upper quartiles *, ** indicate *p* < 0.05 and 0.01, respectively.

The 5-HT content in the cerebral ganglia changed significantly within the four groups [Kruskal–Wallis test for multiple comparisons: H (3, *N* = 48) = 7.94, *p* = 0.0473, Figure 5, the right group of bars]. *Post hoc* tests indicated a significant difference between the E and ER groups, *p* = 0.043).

The 5-HTP content had a similar profile of changes to that of 5-HT [Kruskal–Wallis test: H (3, *N* = 48) = 9.77. *p* = 0.02; Figure 5, the second group of bars]. Namely, a significant increase in the 5-HTP was found in ER group in comparison to the E group after *post hoc* tests (*z* = 3.08; *p* = 0.012).

The 5-HIAA content was significantly higher in all three experimental groups compared to the control [Kruskal–Wallis test: H (3, *N* = 48) = 24.15, *p* = 0.001, Figure 5, the third group of bars, E/Cn *p* = 0.005; ER/Cn *p* = 0.0001; EE/Cn *p* = 0.02].

The Nac-5-HT content was higher in the ER and EE groups compared to the E group [Kruskal–Wallis test: H (3, *N* = 48) = 22. 07, *p* = 0.0001] and as a tendency compared to the control (*post hoc* ER/Cn *p* = 0.054; EE/Cn *p* = 0.058).

### The Effects of Pharmacological Manipulations With the Serotonergic System on Snail Decision-Making Under Uncertainty

#### The Effect of Serotonin Metabolic Precursor 5-Hydroxytryptamine (5-HTP) on Snail Behavior on the Dry Surface (Experiment 3)

**FIGURE 6:**
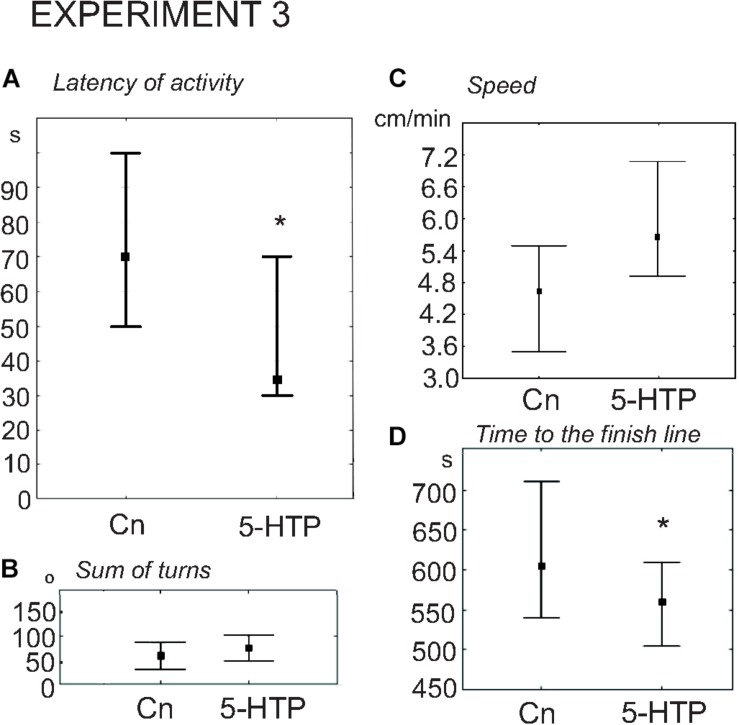
Effects of serotonin precursor 5-HTP on the behavior of snails on a dry surface. **(A)** The latency (in seconds) of the first movement. **(B)** The sum of turns prior to decision-making, in degrees. **(C)** The mean speed of locomotion (in cm/min). **(D)** Time to the finish line (crossing of the virtual arena border) in seconds. The significance of differences was tested using the Mann–Whitney *U*-test. All values are given as median with the lower and upper quartiles * indicates *p* < 0.05.

5-HTP is known to increase the speed of locomotion in *Lymnaea* (Pavlova, 2001, 2019; Tsyganov et al., 2004). Here we found that the snails immersed in 5-HTP (*n* = 27) demonstrated a more rapid onset of activity compared to the control group (*n* = 27). The delay of the first movement was significantly smaller in the experimental snails (Figure 6A, *z* = 2.05, *p* = 0.03, Mann–Whitney *U*-test). There were no differences in the rotational behavior, which is considered to be the orienting phase of the behavior under uncertainty, preceeding the decision-making process. The mean total rotation did not differ between the two groups (*z* = 0.4, *p* = 0.67, Mann–Whitney *U*-test, Figure 6B). The mean speed of locomotion was faster (*z* = 2.09, *p* = 0.03, Mann–Whitney *U*-test, Figure 6C), and the 5-HTP-treated snails reached the border of the arena significantly faster (Figure 6D, *z* = 2.47, *p* = 0.013, Mann–Whitney *U*-test). The ratio of light/dark choice was similar in both groups.

#### The Effect of Serotonin on Snail Behavior on the Dry Surface (Experiment 4)

**FIGURE 7:**
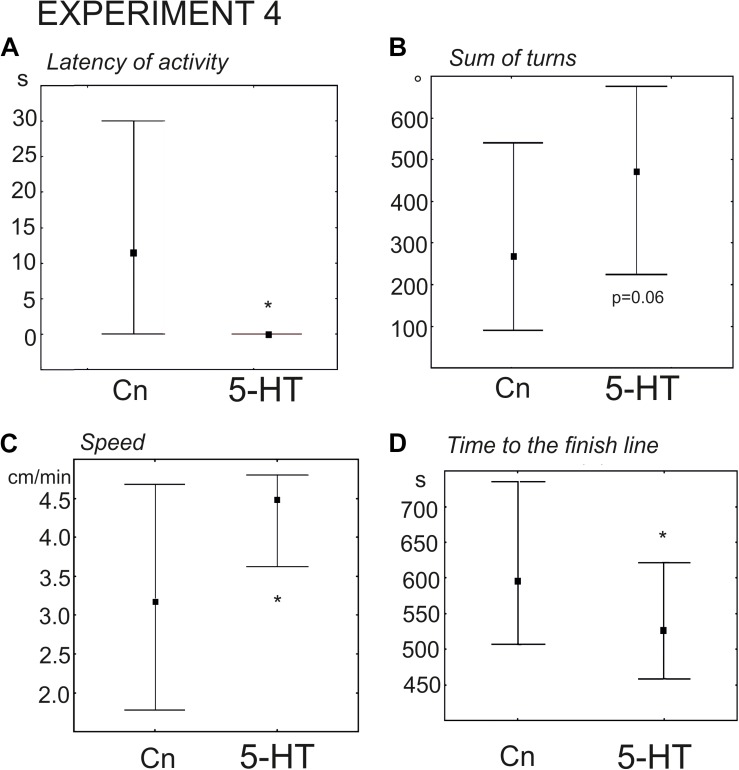
Effects of serotonin (5-HT) on the behavior of snails on a dry surface. **(A)** The latency (in seconds) of the first movement. **(B)** Total rotation prior to decision-making, in degrees. **(C)** The mean speed of locomotion (in cm/min) measured during the phase of the directional crawl (6–8 min from the beginning of the experiment). **(D)** Time to the finish line (crossing of the virtual arena border) in seconds. The significance of differences was tested using the Mann–Whitney *U*-test. The significance of differences was tested using the Mann–Whitney *U*-test. All values are given as median with the lower and upper quartiles * indicates *p* < 0.05.

The snails of the experimental group were immersed in 0.1 mM serotonin (*n* = 24 versus *n* = 30 in the control group). The delay in the first movement was significantly smaller (*z* = 2.54, *p* = 0.01, Mann–Whitney *U*-test, Figure 7A), and the increase in the rotational behavior only achieved a p value equal to 0.06 (Figure 7B). The mean velocity achieved a p value equal to ca. 0.055. Nevertheless, the snails pretreated with serotonin reached the border of the arena significantly faster (*z* = 1.96; *p* = 0.0049, Mann–Whitney *U*-test). We hypothesized that there were no statistically significant differences in the mean speed (*p* = 0.055), because 5-HT- treated snails tended to spend more time in the orienting phase of the behavior (performed more turns). To check this possibility we measured the mean velocity during the phase of the directional crawl (6–8 min after the start of the experiment). It was indeed significantly higher than in the control group (*z* = 2, *p* = 0.03, Mann–Whitney *U*-test, Figure 7C). This finding explains why 5-HT treated snails reached the border of the arena more quickly (Figure 7D). The ratio of light/dark choice was the same for both groups.

#### The Effect of Serotonin Receptor Antagonist Ketanserin on Snail Behavior on the Dry Surface (Experiment 5)

**FIGURE 8:**
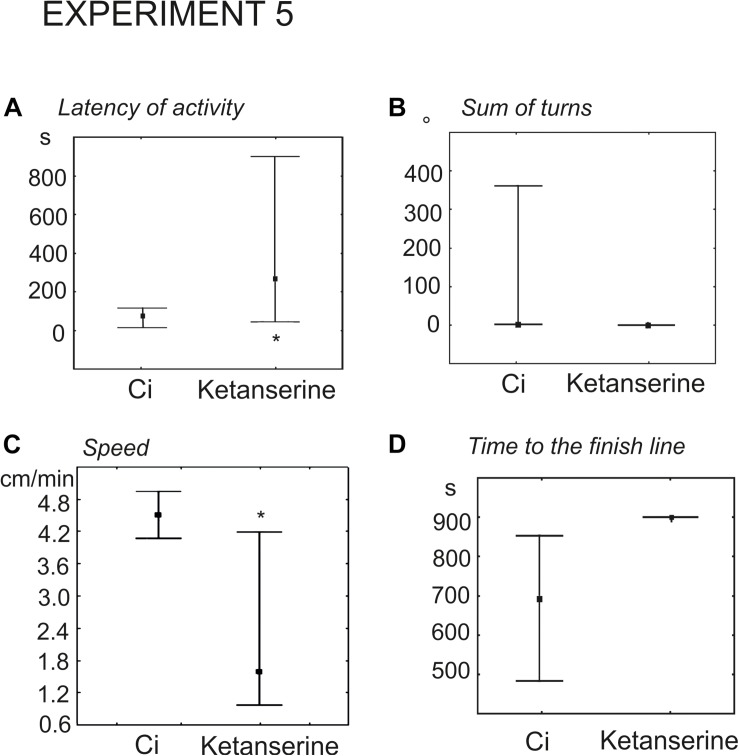
Effects of the serotonin receptor antagonist ketanserin 0.1 mM on the behavior of snails on a dry surface. **(A)** The latency (in seconds) of the first movement. **(B)** The sum of turns prior to decision-making, in degrees. **(C)** The mean speed of locomotion (in cm/min). **(D)** Time to the finish line (crossing of the virtual arena border) in seconds. The significance of differences was tested using the Mann–Whitney *U*-test. All values are given as median with the lower and upper quartiles * indicates *p* < 0.05.

Experimental snails received injection of 100 μl physiological solution containing 0.02 mM (*n* = 9) or 0.1 mM (*n* = 13) ketanserin 15 min prior to the test. The control groups (*n* = 9 and *n* = 13, respectively) received 100 μl of physiological solution. Snails, injected with 0.02 mM ketanserin demonstrated the tendency for later onset of activity (93 ± 32 s versus 28 ± 12, *z* = 1.9, *p* = 0.051, Mann Whitney U test). The effect was significant in the group that received 0.1 mM ketanserin (424 ± 110 versus 78 ± 20, *z* = 2.1, *p* = 0.035, Mann–Whitney *U*-test, Figure 8A).

There were no differences in the mean total rotation between the 0.02 mM ketanserin group and the control group (*z* = 0.68, *p* = 0.49, Mann–Whitney *U*-test). Higher concentration of ketanserin decreased total rotation (*z* = 1.9, *p* = 0.049, Figure 8B).

The overall speed of locomotion was significantly lower in snails treated with 0.02 and 0.1 mM ketanserin than in the control groups (*z* = 2.0, *p* = 0.045 and *z* = 2.2, *p* = 0.027, respectively, Mann–Whitney *U*-test (Figure 8C).

**FIGURE 9:**
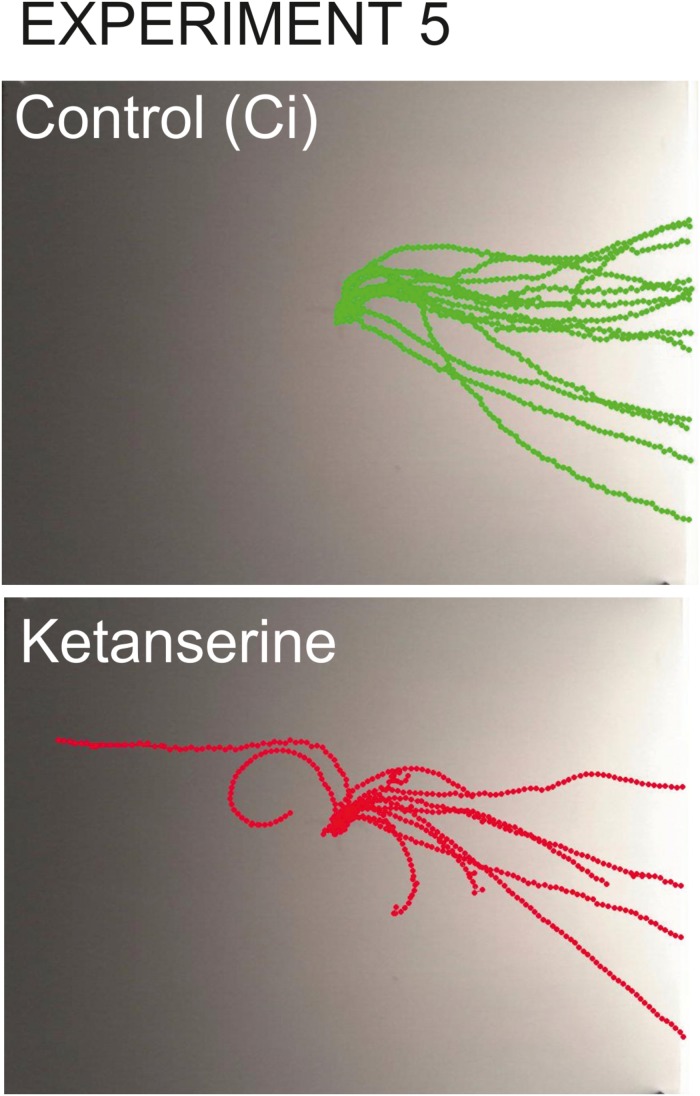
Overlay tracks of snails from the control group injected with saline and the experimental group injected with 0.1 mM ketanserin. In the group injected with ketanserin (lower image, red tracks), the intermediate direction choices (semi-circular routes) can be seen which never occurred in any other groups. The tracks of experimental snails are also obviously shorter since the speed of locomotion was lower in ketanserin-treated animals.

The experimental snails reached the border of the arena later, if ever. The time to the finish line was 808 ± 51 s and 837 ± 45 s in the 0.02 and 0.1 mM ketanserin in contrast to the 556 ± 32 s and 680 ± 55 s in respective controls (*z* = 2.85, *p* = 0.004 and *z* = 2.29, *p* = 0.02, Figures 8D, 9).

In the snails injected with ketanserin, the choices of intermediate directions (semi-circular routes) were observed (*n* = 5), which never occurred in any other groups (Figure 9).

## Discussion

The role of serotonin in the immediate and delayed effects of physical exercise has been intently studied in mammals (Kondo and Shimada, 2015; Heijnen et al., 2016). The serotonergic system is linked with the activation of neurotrophic factors, increased neurogenesis, improved emotional state and memory after exercise. Interestingly, in a quite distant zoological taxon, mollusks, serotonin is involved in the control of locomotion on the one hand (Kabotyanski et al., 1990; Satterlie, 1995; Pavlova, 2001, 2019; Gillette, 2006) and modulates cognitive traits such as memory on the other (Benjamin et al., 2000; Balaban, 2002; Balaban et al., 2016; Deryabina et al., 2018; Nikitin et al., 2018; Totani et al., 2019). Recently, immediate effects of intense locomotion on decision-making under uncertainty were reported in the snail *Lymnaea stagnalis*, suggesting a possible link between motor and cognitive functions of serotonin in mollusks (D’yakonova, 2014; Korshunova et al., 2016). However, there are substantial differences between mammals and mollusks in the structure of their serotonergic systems. One of the most prominent ones is the opposite functioning of serotonin autoreceptors, which are inhibitory in mammals and excitatory in mollusks (Gillette, 2006; Dyakonova et al., 2009). Whether serotonin mediates the behavioral effects of intense locomotion in *Lymnaea* despite these differences remains to be explained.

We demonstrated that intense locomotion has delayed effects on decision-making in *Lymnaea*. Previous exercise manifests itself not only in the electrical properties of serotonergic neurons as reported earlier (Korshunova et al., 2016; Dyakonova et al., 2019) but also in the altered metabolism of serotonin. Immediate and delayed effects are directly manifested in the metabolism in the serotonergic neurons related to locomotion. Moreover, serotonin metabolism appears to be regulated differently in different ganglia of the snail. Remarkably, the most prominent changes were detected in the rest-after-exercise group. Pharmacological manipulation of serotonin content and receptor availability (Table 1) suggests that serotonin is likely to be responsible for the general acceleration of behavior in the conditions of uncertainty immediately after the exercise. This effect can be explained by the serotonin influence on the motor activity (faster onset of movements and higher speed of locomotion). However, our results clearly show that the decreased number of turns during orienting behavior, caused by preceding motor activity and resulting in the facilitation of decision-making, can not be attributed to serotonin.

**TABLE 1:** The effects of behavioral and pharmacological treatments on behavior of snails in a novel environment (asymmetricaly lit dry arena).

Procedure (source)\parameter	Latency of movement	Performance of turns (orienting phase)	Locomotion speed	Time to the finish line
Exercise 2 h (Korshunova et al., 2016)	Decreased (Korshunova et al., 2016)	Decreased (Korshunova et al., 2016)	Increased (Korshunova et al., 2016)	Decreased (Korshunova et al., 2016)
Exercise 2 h and rest 2 h (Dyakonova et al., 2019; here, Experiment 1)	No change (Figure 2A)	Decreased (Figures 2B,C)	No change in the mean speed (Figure 2C, locomotor arousal develops faster (Figure 3D; see also Dyakonova et al., 2019).	No change (Figure 2D)
Treatment with serotonin precursor 5-HTP (here, Experiment 3)	Decreased (Figure 6A)	No change (Figure 6B)	Increased (Figure 6C)	Decreased (Figure 6D)
Treatment with serotonin (here, Experiment 4)	Decreased (Figure 7A)	No change (Figure 7B)	Increased as tendency (Figure 7C, *p* = 0.055)	Decreased (Figure 7D)
Treatment with the 5HT_2_ antagonist, ketanserine (here, Experiment 5)	Increased (Figure 8A)	Decreased (Figure 8B)	Decreased (Figure 8C)	Increased (Figure 8D)
Stress by shaking 2 h (here, Supplementary Material)	No change (Supplementary Figure S1A)	No change (Supplementary Figure S1B)	Decreased (Supplementary Figure S1C)	Increased (Supplementary Figure S1D)

### Intense Locomotion and/or Stress?

Shallow water is not an artificial situation for *Lymnaea* ecology as the pond snails can be observed crawling in low water in their natural habitats. Such crawling is a particular type of locomotion, a muscular one, because ciliary locomotion is not effective enough in such conditions. However, low water is not their optimal niche, and, in addition, it is also unusual for the snails kept as laboratory stock. We have discussed the possible involvement of stress in the effects of intense locomotion in our previous paper (Korshunova et al., 2016). Indeed, mild stress unavoidably accompanies intense physical load in natural and experimental conditions in animals, including mammals and humans. Some signatures of mild stress, such as cognitive arousal and resistance to threatening stimuli resemble the effects of motor load in mammals as well as in snails (Kavaliers, 1987; Heijnen et al., 2016). Numerous observations associate serotonin with stress-induced behavior in vertebrates and invertebrates (Shartau et al., 2010; Bacqué-Cazenave et al., 2017; Rillich and Stevenson, 2018). On the other hand, the behavioral and neurochemical effects differ between the exercise-induced stress and stress due to “negative life events” in mammals (Heijnen et al., 2016).

In *Lymnaea*, we found that novelty-induced stress does not explain increased locomotion (Korshunova et al., 2016 and present data with the Cn control group). We also knew (an unpublished observation) that severe acute stressors, such as nociceptive stimuli, have consequences opposite to those of intense locomotion, increasing the latency of motor activity in the arena. Recently, we finished an experiment in *Lymnaea* imitating another natural stressful situation, turbulent water (Supplemenatry Material). It was induced by 2 h shaking with the frequency of 200 cycles per min., which prevented snails from gliding and crawling. The effect of this treatment on subsequent behavior on the dry surface was highly significant, but it was not at all similar to the exercise effect (Supplementary Figures S1A–E). Intense shaking decreased the speed and increased the time to the finish line, with no changes in the performance of turns and the delay of the initial movement.

Therefore, it seems that the consequences of stress in *Lymnaea* depend on the procedure used to induce it. In these circumstances, we still do not exclude the involvement of mild stress effects in the effects of intense locomotion but, however, prefer to describe the exact behavioral treatment rather than use a vague notion of “stress”.

### The Delayed Effects of Intense Locomotion and the Preadaptation to Novelty Hypothesis

In mammals, including humans, physical activity has long-lasting behavioral and cognitive effects that have attracted the attention of researchers as being beneficial and even curative (Heijnen et al., 2016). The similarity of the behavioral effects of exercise in animals of various phyla prompted us to formulate a general hypothesis on the possible biological benefits of these effects. The hypothesis suggests that: (1) The immediate effects of the exercise can be beneficial for the acceleration of decision-making during fast locomotion to cope with rapid changes in the environment; (2) The delayed behavioral changes are a manifestation of the feedforward preadaptation to a less familiar environment, which animals are likely to face following a period of intense locomotion. Preadaptation means the possession of certain characteristics by an organism which make it more adaptable to a future environmental changes. The new environment means high uncertainty, low predictability of events and excess of new information. These are serious challenges for the animal, which reduce the chances of its survival, and therefore preadaptation to possibly new conditions seems biologically justified.

The known effects of exercise, such as facilitation of learning and memory for novel information (Epp et al., 2016), activation of neurogenesis (van Praag et al., 1999, Lee et al., 2013), enhancement of goal-oriented behavior and “effortfulness” (Laurence et al., 2015, Korshunova et al., 2016), decreasing the sensitivity to external disturbances and stress (Stevenson et al., 2005) and facilitation of effort-based decision-making (Bernacer et al., 2019) agree with this hypothesis. These behavioral and physiological changes may help the organism to cope with excess of novel information and stress.

To the best of our knowledge, this is the first study to demonstrate the delayed effects of intense locomotion on the behavior under uncertainty in an invertebrate, the mollusk *Lymnaea stagnalis*. The delayed effects of exercise were detected in this animal at both the behavioral and the metabolic levels. In our behavioral paradigm, the delayed effect of intense crawling was a decreased number of orienting turns in the first phase of snail behavior in the novel environment. This parameter depends on the level of uncertainty in the environment (Korshunova et al., 2016), therefore we suggest that a decreased performance of turns can be considered as a facilitation of the decision-making process. This effect had been demonstrated in our previous study as an immediate result of intense locomotion (Korshunova et al., 2016), and it can now be extended to the delayed effects of the exercise. The persistence of this effect is contrasted by the disappearance of changes in the latency and speed of locomotion in the novel environment after rest. The mean speed of locomotion of the ER snails was significantly increased compared to the intact control (Ch) group, but not compared to snails kept in novel conditions (Cn), in contrast to those that were tested immediately after exercise (Korshunova et al., 2016). Nevertheless, faster locomotor arousal was observed in ER snails (Dyakonova et al., 2019). The illustration provided here (Figure 3D) agrees with this earlier finding.

Intense locomotion affected the process of decision making (the total rotation and, hence, the delay of the transfer from rotational to goal-oriented behavior) but not the result, the choice between the light and dark sides of the arena. For a snail, which has a very low speed of locomotion, in the situation of limited time and high uncertainty, long exploration of the environment with frequent changes of direction is hardly the best strategy. Investing time and energy into crawling in the chosen direction will make the survival more probable. Preadaptation, as we propose, increases the level of confidence in ambiguous situations (when animals need to solve problems that have no clear or defined answers) (Vorontsov and Dyakonova, 2017).

The observed behavioral effects of past exercise are in line with electrophysiological data obtained from the pedal serotonergic neurons PeA controlling locomotion in *Lymnaea* (Dyakonova et al., 2019). Rest after exercise eliminates the excitatory effect of exercise on the activity of these neurons in the CNS, but not in completely isolated neurons. In other words, serotonergic neurons remain in the internally excited state under external network inhibition in animals that rest after the terrestrial locomotion. This ambiguous state may underlay faster transition from the aquatic to terrestrial locomotion.

### Differential Regulation of Serotonin Metabolism During Exercise and Rest

Exercise causes dynamic fluctuations in the content of serotonin in the mammalian brain, which may change in opposite directions in some brain regions (Heijnen et al., 2016). In *Lymnaea*, the sensitivity of serotonin metabolism to exercise is shown in our experiments performed on four groups of snails with different experience of intense locomotion: crawling for 2 or 4 h and 2 h of crawling followed by 2 h of rest. In general, the results suggest an elevation of serotonin metabolism in response to exercise. Interesting details are identified regarding site-specificity and time-course of this effect.

Thus, we demonstrated that the level of serotonin metabolites changes differently, in response to intense locomotion, in the cerebral compared to the pedal ganglia. The cerebral and pedal ganglia are functionally different. The pedal ganglia are strongly involved with the control of locomotion and respiration, while the cerebral ganglia seem to play part in the modulation of various forms of behavior, from feeding to learning. In the cerebral ganglia, the higher level of serotonin content was observed in the rest-after-exercise group (statistically significant in comparison to the exercise group and as a tendency in comparison to the control one). By contrast, in the pedal ganglia, the content of serotonin did not statistically change in the ER group (and it tended to be lower than in other groups). Therefore, serotonin metabolism seems to be differently regulated in different parts of the CNS in mollusks. Presumably, the change in distribution helps to cope with different needs for serotonin by different neuronal ensembles in various behavioral contexts.

The absence of differences in the content of serotonin in the pedal ganglia in the ER group compared to control was accompanied by a 10-fold increase in the 5-HTP concentration and a significant increase in the content of both catabolites of serotonin. It is likely that the increased catabolism of serotonin and the termination of its synthesis could be responsible for the effects of rest described above. Our data point to aromatic amino acid decarboxylase (AAD) activity as the main source of the observed changes. It is likely that transient inhibition of AAD in the rest-after-exercise group was responsible for the accumulation of 5-HTP. This possibility can be experimentally verified in the future.

Several other studies have suggested that the AAD activity is an additional source of regulation in the metabolism of monoamines. Independent regulation of 5-HT and 5-HTP content and similar accumulation of 5-HTP had been identified in the CNS of *Lymnaea* earlier, specifically, after food deprivation (Aonuma et al., 2016). Potentiation of L-DOPA effects in the presence of high dopamine concentration similarly suggests that AAD is inhibited by dopamine (Dyakonova et al., 2009).

One interesting result of our HPLC analysis was that the strongest changes in the serotonin precursor and catabolites were found in the snails that rested for 2 h after intense crawling. Therefore, rest after exercise produces highly specific effects on serotonin metabolism that are not identical to simple vanishing of exercise effects or compensatory synthesis.

### Serotonin and Behavioral Changes Caused by Preceding Intense Locomotion in Lymnaea

Serotonin appears to be the strongest candidate for the evolutionary conserved neuromodulator that mediates the behavioral effects of exercise. It remained, however, unknown to what extent serotonin can mimic the behavioral effects of exercise in *Lymnaea*.

We addressed this question by testing the effects of serotonin precursor, serotonin and the serotonin receptor antagonist ketanserin on snail behavior on the dry, asymmetrically lit arena. In this behavioral test, the immediate and delayed effects of exercise were described in detail (Table 1). The immediate effects of exercise were characterized by the reduced latency of the first movement, the reduced number of orienting turns, faster decision-making and higher speed of locomotion (Korshunova et al., 2016). The delayed effects described here were also characterized by the reduced number of turns and faster locomotor arousal, with only weak differences in the mean speed of locomotion.

The effects of serotonergic drugs investigated here (Table 1) are in concordance with each other and suggests certain conclusions about the role of serotonin in the behavioral effects of intense locomotion. Serotonin and its metabolic precursor decreased the latency to start movement in a novel arena. This effect was similar to the effect of immediate exercise. 5-HTP, similarly to exercise, decreased the time of the onset of the movement and the time it took to reach the virtual border of the arena. Serotonin produced similar but weaker changes. We suggest that the difference in the effectiveness of the two drugs can be explained by different mechanisms of their action on the serotonin receptors. There is no evidence that 5-HTP has any direct effect on serotonin receptors. The serotonergic neurons first uptake it, then metabolize into the serotonin by AAD. 5-HTP acts via the enhanced release of serotonin at the conventional sites of serotonin release. In contrast, incubation in serotonin may result in less specific action on various serotonin and monoamine receptors. 5-HTP is more effective than serotonin in many other experimental models: a variety of gastropod mollusks (for review, see Sakharov, 1991); crickets (Ureshi et al., 2002; Dyakonova and Krushinsky, 2013; Rillich and Stevenson, 2018); locust (Anstey et al., 2009). Neither serotonin in any concentration nor its metabolic precursor affected the orienting phase of the behavior (the rotation prior to decision-making). The serotonin antagonist ketanserin increased the latency to start movement, reduced the number of turns, and strongly suppressed locomotion (Table 1).

The data suggest that serotonin can mediate locomotor arousal in the exercise effects but is not responsible for the reduction in the length of the orienting phase. Therefore, the neurochemical mechanism responsible for faster decision-making after exercise in *Lymnaea* remains to be found.

This suggestion is in line with the effects of serotonergic drugs in mollusks reported earlier. Activation of the aquatic and terrestrial locomotion in *Lymnaea stagnalis* by serotonin or 5-HTP was found in several studies (Pavlova, 2001, 2019; Tsyganov et al., 2004). Activation of searching movements by serotonin was shown in the terrestrial snails *Helix* (Roshchin and Balaban, 2012) and *Cepaea* (Dyakonova et al., 1995). In *Lymnaea stagnalis*, serotonin precursor also promoted approaching unfamiliar objects as well as orienting turns in response to tactile stimuli of tentacles (D’yakonova and Sakharov, 1995). The possible influence of intense locomotion on cognitive functions like learning, memory and predictive abilities has not yet been investigated in mollusks. Therefore, serotonin, although it does not seem to be involved in the facilitation of decision-making after the exercise, might still influence other cognitive functions.

In several models where the central and peripheral effects of serotonin were elucidated, a notable agreement between them had been found (Sakharov, 1990; Satterlie, 1995; Gillette, 2006; Roshchin and Balaban, 2012; Pavlova, 2019). These findings support the hypothesis of the integrative function of serotonin (Sakharov, 1990), which implies that, in relatively simple organisms, the effects of the neurotransmitter on various targets and receptors may serve the same integrative function. This view later received important elaborations (Gillette, 2006; Harris-Warrick and Johnson, 2010). The first approach suggests that neurotransmitter’s synergistic effects can be seen within the one functional system, while the switching between the functional systems (for example between escape and feeding behavior, both of which are controlled, in mollusks, by serotonin) relies on competitive interactions between central neurons (Gillette, 2006). The second review collected and analyzed examples of neurotransmitter’s antagonistic effects within the same functional group, and suggested the role for these antagonistic interactions in fine-tuning of neuromodulator’s effects (Harris-Warrick and Johnson, 2010). One needs to keep these complexities in mind when we discuss the effects of neurotransmitters applied through a bath, the procedure used in our investigation.

We have discussed earlier that it is difficult to specify whether serotonin exerts its behavioral effects as a synaptic neurotransmitter, an extrasynaptic neurotransmitter, a neuromodulator, a neurohormone or a metamodulator (Dyakonova et al., 2015). We believe that this difficulty is only partially explained by the absence of empirical details. The modern classification of neuronal signal molecules is based on the outdated conception that a neurotransmitter only transmits the electrical events between the synaptically connected neurons, while the very same chemical substance, spilled over the synapse or involved in volume transmission, is called a neuromodulator. Instead, all neurochemical events, from synaptic to hormonal, can be put on a single scale, thus avoiding the redundant classification of neuroactive substances and at the same time allowing for a better explanation of empirical data.

We can conclude that the results of our behavioral, pharmacological and biochemical experiments suggest that serotonin is likely to play a role in the effects of exercise on the *Lymnaea* behavior in a novel environment. However, it appears to be responsible for the locomotor activation and the increase in the intensity of the whole behavioral program in the novel environment, having no effect on the structure of this behavior. Neuromodulators that control rotational searching behavior and are responsible for faster cessation of the orienting phase (facilitation of decision-making) after exercise remain to be found in *Lymnaea*. Therefore, the effects of exercise on the facilitation of decision-making are likely to be mediated by multitransmitter neuromodulatory control in mollusks.

## Data Availability Statement

The datasets generated for this study are available on request to the corresponding author.

## Ethics Statement

This study was carried out in invertebrate animals, the pond snails Lymnaea stagnalis, in accordance the protocol has been approved by the responsible authorities of our institutions.

## Author Contributions

HA performed HPLC experiments and edited the manuscript. MM performed behavioral experiments (delayed effects of locomotion, 5-HT and supplementary stress experiment). BB performed behavioral experiments (5-HTP and ketanserin effects). YT performed behavioral experiments and made dissections for HPLC analysis. DV performed analysis of behavioral data using the EthoVision software. IZ participated in behavioral experiments and discussed results. EI analyzed the results and edited the manuscript. VD planned the experiments and wrote the present manuscript.

## Conflict of Interest

The authors declare that the research was conducted in the absence of any commercial or financial relationships that could be construed as a potential conflict of interest.
