# Supplementary material for: The Role of Serotonin in the Influence of Intense Locomotion on the Behavior Under Uncertainty in the Mollusk Lymnaea stagnalis
Source: Front Physiol. 2020 Mar 17;11:221. doi: 10.3389/fphys.2020.00221 (PMC7091490; doi:10.3389/fphys.2020.00221)
Supplement: Supplementary file 1 [file Table_1.docx]

Supplement to the paper:

**The role of serotonin in the effects of intense locomotion on behavior under uncertainty in the mollusk *Lymnaea stagnalis***

***Hitoshi Aonuma^1,2†^, Maxim Mezheritskiy^3†^, Boris Boldyshev^4^, Yuki Totani^5^, Dmitri Vorontsov^3^, Igor Zakharov^3^, Etsuro Ito^5^, Varvara Dyakonova^3^****

Supplementary experiment: Effects of stress, induced by shaking, on subsequent behavior on a dry arena in *Lymnaea stagnalis*.

Methods:

Stress was evoked by 2 hours shaking with frequency 200 shakes per min, which prevented snails from gliding and crawling. Control snails were kept in deep water (300 ml containers) so they could use ciliary locomotion in similar light conditions. The experimental (n=38) and control animals (n=38) were investigated in random order in a single experiment.

Results

The latency of the first movement did not differ (z=0.6, p=0.5, Mann Whitney U test, **Supplementary Figure 1A**), there was not difference in the rotational behavior (z=1.2, p=0.2, Mann Whitney U test, **Supplementary Figure1B**), the mean velocity was significantly lower (z=1.96; p=0.0049, Mann-Whitney U test), , **Supplementary Figure 1C).** Turbulent shaking increased the time to the finish line (z=3.3, p=0.0008, **Supplementary Figure 1D**).


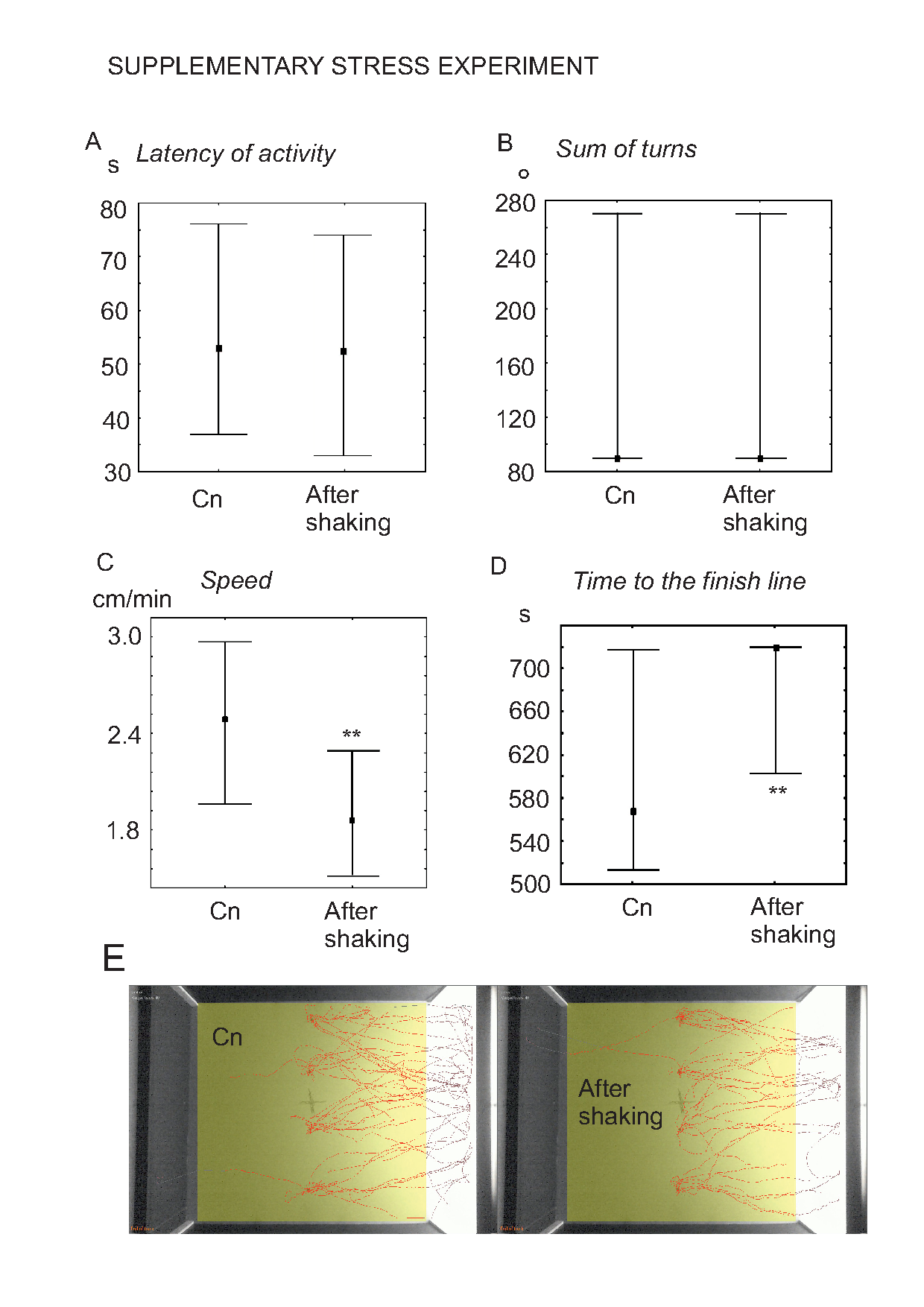
**Figure 1.** Effects of stress, induced by shaking, on subsequent behavior on a dry arena in *Lymnaea stagnalis*. **(A)** The latency (in seconds) of the first movement. **(B)** The sum of turns prior to decision-making, in degrees. **(C)** The mean speed of locomotion (in cm/min). **(D)** Time to the finish line (crossing of the virtual arena border) in seconds. The significance of differences was tested using the Mann-Whitney U test. All values are given as median with the lower and upper quartiles *, ** indicate p<0.05 and 0.01, respectively. **(E)**  Overlay tracks of snails from the control and experimental (stressed) group.
